# Glaucine inhibits breast cancer cell migration and invasion by inhibiting MMP-9 gene expression through the suppression of NF-κB activation

**DOI:** 10.1007/s11010-015-2339-9

**Published:** 2015-02-11

**Authors:** Hyereen Kang, Sung-Wuk Jang, Jhang Ho Pak, Sungbo Shim

**Affiliations:** 1 Department of Biomedical Sciences, University of Ulsan College of Medicine, Seoul, 138-736 Republic of Korea; 2 Department of Biochemistry and Molecular Biology, University of Ulsan College of Medicine, Seoul, 138-736 Republic of Korea; 3 Asan Institute for Life Sciences, University of Ulsan College of Medicine, Asan Medical Center, Seoul, Republic of Korea; 4 Neuromarker Resource Bank (NRB), Seoul, 138-736 Republic of Korea

**Keywords:** Invasion, Migration, MMPs, Natural compound, Breast cancer

## Abstract

Matrix metalloproteinase-9 (MMP-9) plays a central role in the invasion and metastasis of various types of cancer cells. Here, we demonstrate that glaucine, an alkaloid isolated from the plant* Corydalis turtschaninovii* tuber (Papaveraceae), can inhibit the migration and invasion of human breast cancer cells. We further show that glaucine significantly blocks phorbol 12-myristate 13-acetate (PMA)-induced MMP-9 expression and activity in a dose-dependent manner. Results from reporter gene and electrophoretic mobility shift assays revealed that glaucine inhibits MMP-9 expression by suppressing activation of the nuclear transcription factor nuclear factor-κB (NF-κB). Moreover, glaucine attenuates PMA-induced IκBα degradation and nuclear translocation of NF-κB. Finally, we also found that glaucine inhibits invasion and MMP-9 expression in the highly metastatic MDA-MB-231 breast cancer cell line. Taken together, our findings indicate that the MMP-9 inhibitory activity of glaucine and its abilities to attenuate IκBα and NF-κB activities may be therapeutically useful as a novel means of controlling breast cancer growth and invasiveness.

## Introduction

Metastasis is a defining characteristic of malignant cancer cells, and uncontrolled metastasis is the leading cause of death in patients with cancer. Metastasis is a multi-step process involving degradation of the extracellular matrix (ECM), adhesion of cancer cells to endothelial cells, extravasation leading to infiltration of the underlying tissue, and metastatic foci formation [1, 2]. When tumor cells metastasize, a number of proteolytic enzymes contribute to the degradation of ECM components and the basement membrane [3, 4].

Matrix metalloproteinases (MMPs) are a family of zinc-dependent endopeptidases that degrade ECM components and are therefore important in several physiological and pathological functions, including tumorigenesis and cancer metastasis [5, 6]. At least 20 types of MMPs have been identified to date, and MMP-2 and -9 are highly correlated with tumor invasiveness and metastatic potency [7]. Various pathological processes such as tumor invasion, atherosclerosis, inflammation, and rheumatoid arthritis can stimulate MMP-9 synthesis and secretion, whereas MMP-2 is usually constitutively overexpressed [8, 9]. Base on reports from several different laboratories, basal MMP-9 levels in most cancer cell lines are usually low, and its expression can be induced by treatment with cytokines and phorbol 12-myristate 13-acetate (PMA), which activates transcription factors such as nuclear factor (NF)-κB and activator protein (AP)-1 [10]. Thus, MMP-9 is an attractive target to decrease the invasiveness and metastasis of cancers that overexpress this protein.

Glaucine [(*S*)-(+)-1,2,9,10-tetramethoxyaporphine] is an alkaloid isolated from the plant *Corydalis turtschaninovii* tuber (Papaveraceae) that has been used for years as a traditional folk remedy for cough and for its spasmolytic, neuropharmacologic, hypothermic, and blood pressure-lowering activities [11–13]. Glaucine is also known to inhibit the proliferation of a variety of tumor cells, including leukemia, cervical, bladder, breast, and colon cancer cells [14–16]. In addition, glaucine seems to exert chemopreventive properties against melanoma, and it also suppresses tumor migration [17]. However, the molecular mechanism underlying the beneficial effects of glaucine are not yet fully understood. In this study, we investigated how glaucine treatment affected MMP-9 expression in breast cancer cells and explored the underlying upstream signaling mechanisms. We found that glaucine significantly inhibited MMP-9 gene expression by suppressing NF-κB activation, which subsequently reduced the invasion and migratory abilities of human breast cancer cells.

## Materials and methods

### Cells and reagents

The human breast cancer cell line MCF-7 and MDA-MB-231 cells (ATCC, Manassas, VA, USA) were maintained in RPMI 1640 supplemented with 10 % heat-inactivated FBS (HyClone, Logan, UT, USA), penicillin (100 U/ml), and penicillin–streptomycin (100 μg/ml) at 37 °C with 5 % CO_2_ atmosphere in a humidified incubator. Glaucine was obtained from MP Biomedicals Korea (Seoul, Korea). Gelatin was obtained from DIFCO (Lexington, KY, USA). Lipofectamine 2000 reagent was purchased from Invitrogen (Carlsbad, CA, USA). PMA was purchased from Calbiochem (La Jolla, CA, USA). Anti-MMP-9 antibody was purchased from Abcam (Cambridge, United Kingdom). All the chemicals not included above were from Sigma (St. Louis, MO, USA).

### Cell proliferation and viability assay

All proliferation and viability assays were based on the 3-(4,5-dimethylthiazol-2-yl)-2,5-diphenyltetrazolium bromide (MTT) method. Cells were seeded in a 96-well plate at a density of 1 × 10^4^ cells/well. The cells were treated with various concentration of glaucine and allowed to grow for 24 and 48 h. At the end of the experiment, the media was removed and DMSO was added with MTT solubilization solution. Absorbance was measured at 550 nm (SpectraMAX 340PC; Molecular Devices, Sunnyvale, CA, USA).

### Colony-forming assay

MCF-7 cells were seeded into 6-well plate and allowed to attach for 24 h at 37 °C in culture medium. Cells were then treated with various concentration of glaucine. After 4 days, colonies were fixed with fixing solution (methanol:acetic acid = 3:1) for 10 min at room temperature and stained with 0.01 % crystal violet solution. Plates were washed with PBS and were photographed (Olympus Microscope System IX51; Olympus, Tokyo, Japan).

### In vitro invasion assay

Matrigel invasion assays were used to assess the effect of glaucine in MCF-7 or MDA-MB-231 cells. The 8-µm pore-size polycarbonate nucleopore filter inserts in a 24-well transwell chamber (BD Biosciences, San Jose, CA, USA) was coated with 30 µg/well matrigel (Sigma). Glaucine-treated MCF-7 cells were seeded into the upper part of the matrigel-coated filter, and serum-free RPMI with or without 100-nM PMA was added to the lower part. After 36 h, the cells that had migrated through the matrigel and the 8-µm pore-size membrane were fixed, stained, and counted under a light microscope (Olympus Microscope System IX51; Olympus, Tokyo, Japan).

### RNA extraction and semi-quantitative RT-PCR

Total RNA was extracted from cells with Trizol (Invitrogen, Carlsbad, CA, USA) according to the manufacturer’s protocol. Approximately 2 μg of total RNA was used to prepare cDNA using the Superscript First Strand cDNA Synthesis Kit (Bioneer, Daejeon, South Korea). The following primers were used in this study: 5′-TCCCTGGAGACCTGAGAACC-3′ and 5′-CGGCAAGTCTTCCGAGTAGTT-3′ for MMP-9; 5′-CCATCACCATCTTCCAGGAG-3′ and 5′-CCTGCTTCACCACGTTCTTG-3′ for GAPDH. PCR was performed with Platinum Tap polymerase (Invitrogen) under the following conditions: 30 cycles of 96 °C for 40 s, 55 °C (MMP-9) or 60 °C (GAPDH) for 40 s, and 72 °C for 1 min followed by 10 min at 72 °C. The PCR products were electrophoresed on a 2 % (w/v) agarose gel in 1 × Tris–acetate-EDTA (TAE) buffer and stained with ethidium bromide solution.

### Gelatin zymography

The presence of MMP-9 in the supernatants of DMSO or glaucine-treated MCF-7 cells was analyzed with gelatin zymograms. Briefly, cells were incubated in serum-free RPMI and the supernatants were collected after incubation for 24 h, clarified by centrifugation, normalized to the total protein concentration of the cell lysate, mixed with non-reducing Laemmli sample buffer, and separated by electrophoresis in 10 % SDS-PAGE containing 1 mg/ml gelatin (DIFCO). After electrophoresis, gels were re-natured by washing in 2.5 % Triton X-100 solution twice for 30 min to remove all SDS. The gels were then incubated in 50 mmol/L Tris–HCl (pH 7.4), 5 mmol/L CaCl_2_, and 1 μM ZnCl_2_ at 37 °C overnight. After incubation, the gels were stained with 0.05 % Coomassie brilliant blue R-250 for 30 min at room temperature and then destained in distilled water. MMP-9 activities were visible as clear bands on a blue background where the gelatin substrate had been hydrolyzed by enzyme activity.

### ELISA assay

The supernatants were collected for measuring secreted MMP-9 protein. The total MMP-9 protein was assayed according to SensoLyte PlusTM 520 MMP-9 assay system (AnaSpec, San Jose, CA). MMP-9 activity unit was expressed as a change in fluorescence intensity at excitation of 490 nm/emission of 520 nm (SpectraMAX 340PC; Molecular Devices, Sunnyvale, CA, USA).

### Transient transfection and luciferase reporter assay

The transcriptional activities of MMP-9, mNF-κB MMP-9, and NF-κB were measured by luciferase reporter assay using the pMMP-9-Luc, mNF-κB MMP-9, and pNF-κB-Luc reporter plasmids. MCF-7 cells were seeded into 12-well plates. Cells at 70–80 % confluence were co-transfected with 0.2 µg of MMP-9, or NF-κB reporter constructs and 0.2 µg pSV-β-galatosidase for 24 h. The transfected cells were incubated with glaucine and then stimulated with 100 nM PMA for 9 h. Luciferase and β-galactosidase activities were assayed according to the manufacture’s protocol (Promega), using a Luminometer 20/20n (Turner BioSystems, Sunnyvale, CA, United States). Luciferase activity was normalized by β-galactosidase activity in cell lysate and expressed as an average of three independent experiments.

### Cell fraction and immunoblotting

Nuclear and cytosolic factions were prepared using Nuclear and Cytoplasmic Extraction Reagents kit (Fermentas). PCNA and Tubulin were used as markers for nuclear and cytosol proteins, respectively. Lysate proteins were resolved by SDS-PAGE and transferred onto nitrocellulose membranes. The membranes were incubated with TBS containing 0.1 % Tween 20 and 5 % skimmed milk, and then exposed to the desired primary antibodies. After treatment with anti-rabbit or -mouse antibodies conjugated with horseradish peroxidase, the immunoreactive bands were visualized by standard ECL method.

### Electrophoretic mobility shift assay (EMSA)

Double-stranded oligonucleotides containing the NF-κB (5′- AGTTGAGGGGACTTTCCCAGGC-3′) or consensus sequences were 5′-end-labeled with γ−^32^P ATP using T4 polynucleotide kinase. Unincorporated nucleotide was removed by passage over a Bio-Gel P-6 spin column (Bio-Rad, Inc., Hercules, CA). Nuclear extract was incubated with radiolabeled probe for 20 min, and protein-DNA complexes were separated from free probes by electrophoresis on a 4 % native polyacrylamide gel in 0.5 **×** Tris–HCl (pH 7.5), 1 mM MgCl_2_, 50 mM NaCl, 0.5 mM EDTA, 4 % glycerol, 0.5 mM DTT, and 50 mg/ml of poly (dI-dC). Dried gels were visualized by autoradiography.

### Statistics analysis

Statistical analysis was performed by computer program Prism (GraphPad Software, La Jolla, CA). The results are presented as mean ± SE. The statistical significance of differences between groups was analyzed via repeated measures of one-way analysis of variance followed by Student’s *t* test. A *P* value ≤ 0.05 was considered to be significant.

## Results

### Effects of glaucine on the growth of PMA-treated MCF-7 cells

**Fig. 1:**
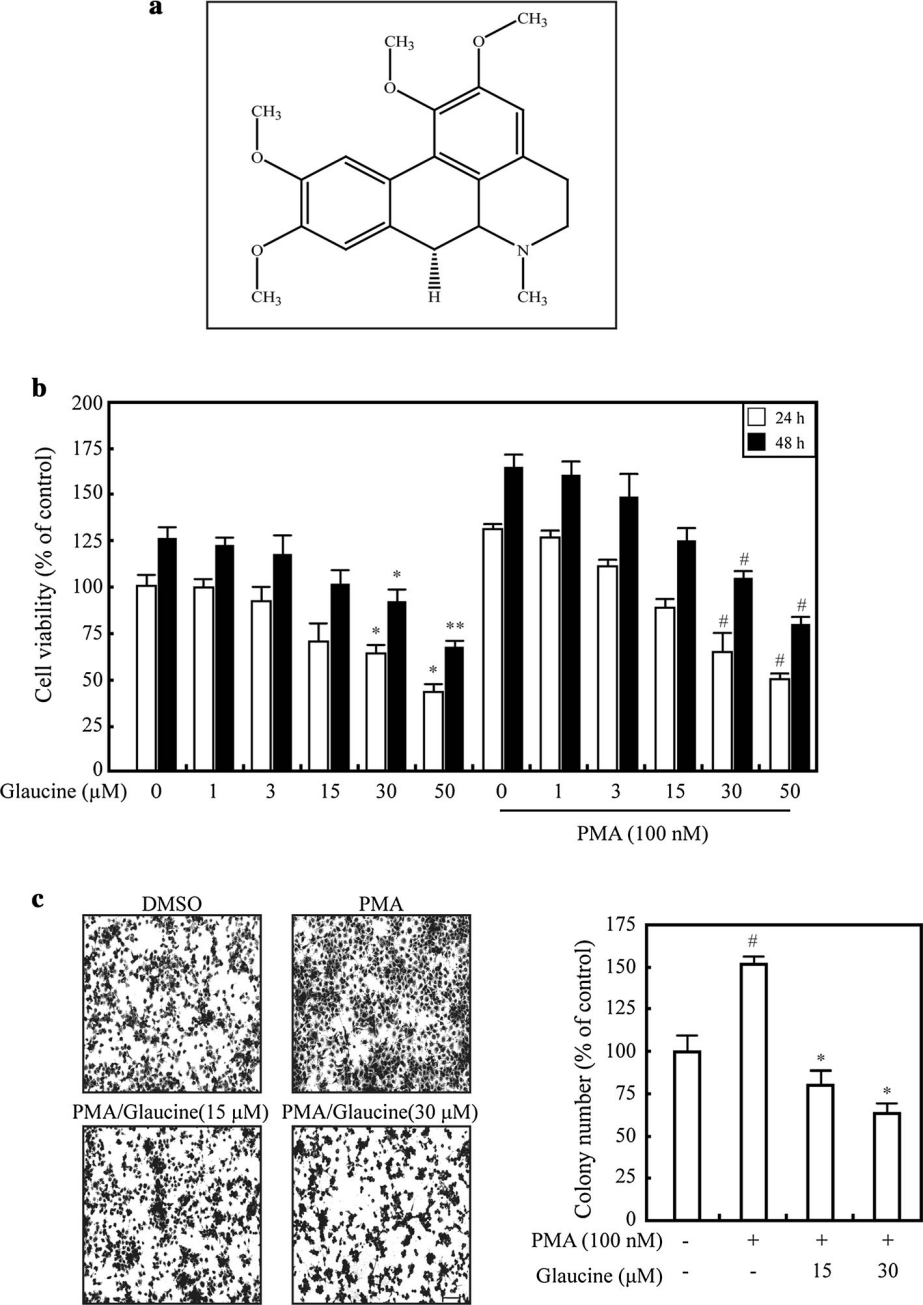
Glaucine suppresses PMA-induced proliferation and invasion of MCF-7 cells. **a** Chemical structure of glaucine. **b** MCF-7 cells were treated with 0–50 μM glaucine in the absence or presence of 100 mM PMA for 24 h (*white bars*) and 48 h (*black bars*), and cell viability was measured using a MMT assay; ^*^ *P* < 0.05, ^**^ *P* < 0.01 versus vehicle alone-treated cells, ^#^ *P* < 0.05 versus PMA alone-treated cells. **c** MCF-7 cells were treated with the indicated concentration glaucine for 30 min and then incubated with 100 nm PMA for 4 days. After 4 days, colonies were fixed with fixing solution for 10 min at room temperature and stained with 0.01 % crystal violet solution. Representative photographs demonstrating colony formation are shown. Original magnification was × 100. *Scale bars*, 100 μm. The number of colonies from triplicate plates was quantified using Image J; ^#^ *P* < 0.01 versus vehicle alone-treated cells, ^*^ *P* < 0.01 versus PMA alone-treated cells

We first examined the effects of glaucine on PMA-induced MCF-7 cell proliferation using MMT assays. Treatment with PMA for 24 and 48 h significantly increased MCF-7 cell viability; however, treatment with >15 μM glaucine decreased cell proliferation in the presence or absence of PMA (Fig. 1b). We performed colony formation assays to further investigate the inhibitory effects of glaucine on cell proliferation. PMA markedly enhanced the colony-forming ability of MCF-7 cells compared to untreated cells (Fig. 1c). In contrast, glaucine at 15 and 30 μM inhibited PMA-induced colony formation by 48 and 63 %, respectively (Fig. 1c).

### Effects of glaucine on migration and invasion in PMA-treated MCF-7 cells

**Fig. 2:**
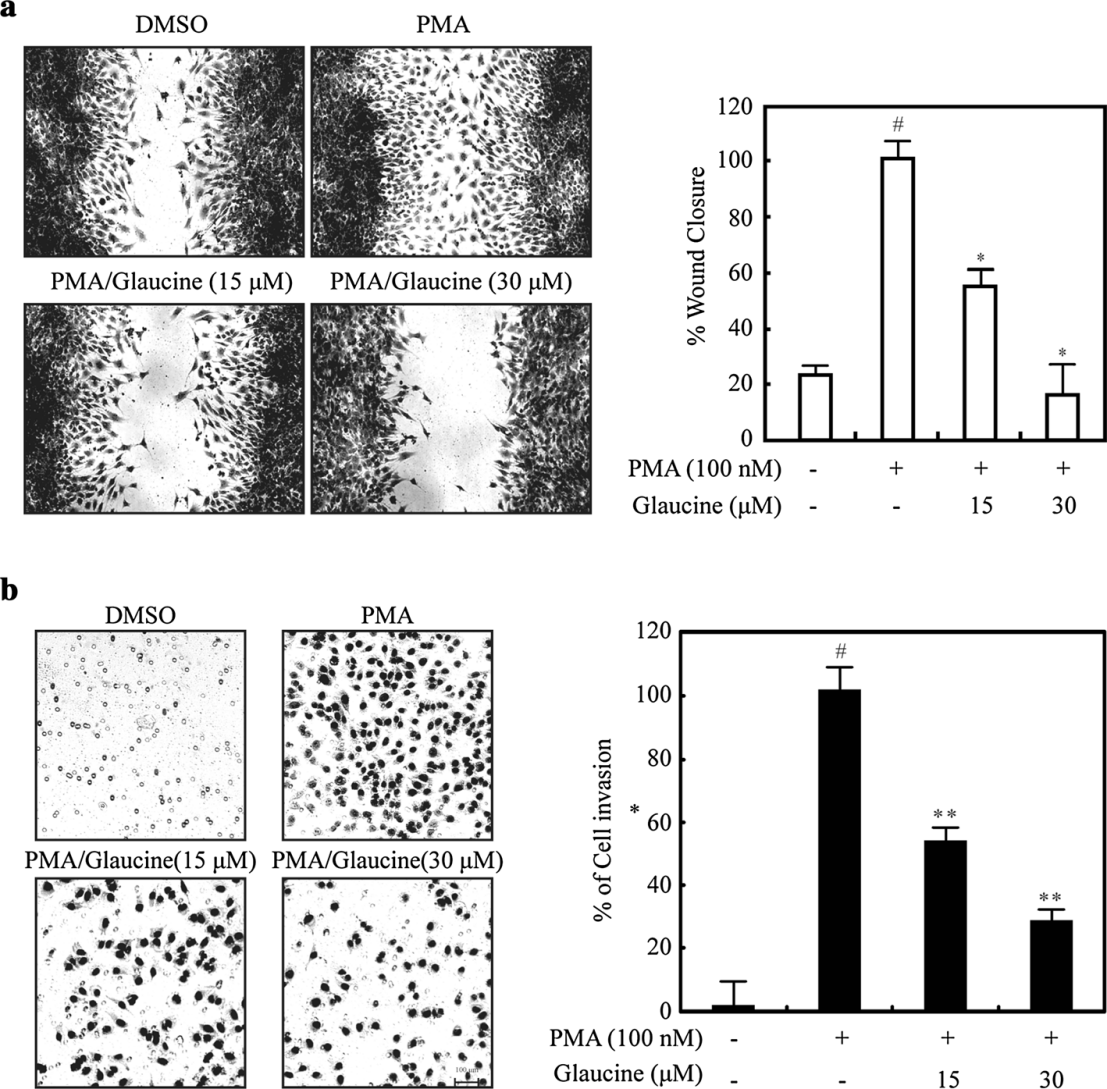
Glaucine inhibits the migration and invasion of PMA-treated MCF-7 cells. **a** PMA-induced MCF-7 cells were scratched with a pipette tip and incubated with the indicated concentration of glaucine for 36 h. The wound area was quantified by measuring the cell-free area. Original magnification was × 100. *Scale bars*, 100 μm. Cell migration into the wound area is represented as the percentage of recovery relative to 0 h. ^#^ *P* < 0.01 versus vehicle alone-treated cells, ^*^ *P* < 0.01 versus PMA alone-treated cells. **b** MCF-7 cells were incubated with 100-nM PMA and the indicated concentration of glaucine for 36 h. The invasion ability of MCF-7 cells was determined by a Matrigel invasion assay. Original magnification was × 200. *Scale bars*, 100 μm. The cell invasion ability was quantified; ^#^ *P* < 0.01 versus vehicle alone-treated cells, ^*^ *P* < 0.01 versus PMA alone-treated cells. Data represent the mean ± SE of three independent experiments

Cell migration is a critical process during many stages of cancer cell metastasis [18]. We, therefore, determined whether glaucine inhibited the invasive behavior of breast cancer cells. As shown in Fig. 2a, glaucine significantly suppressed the migration of PMA-treated MCF-7 cells. To further explore the effect of glaucine on invasion, PMA-induced cells were treated with glaucine (15 or 30 μM), and cell invasion was determined after 36 h. As shown in Fig. 2b, glaucine markedly decreased PMA-induced MCF-7 cell invasion in a dose-dependent manner, indicating that glaucine is an effective inhibitor of breast cancer cell migration and invasion.

### Effects of glaucine on PMA-induced expression and proteolytic activity of MMP-9

**Fig. 3:**
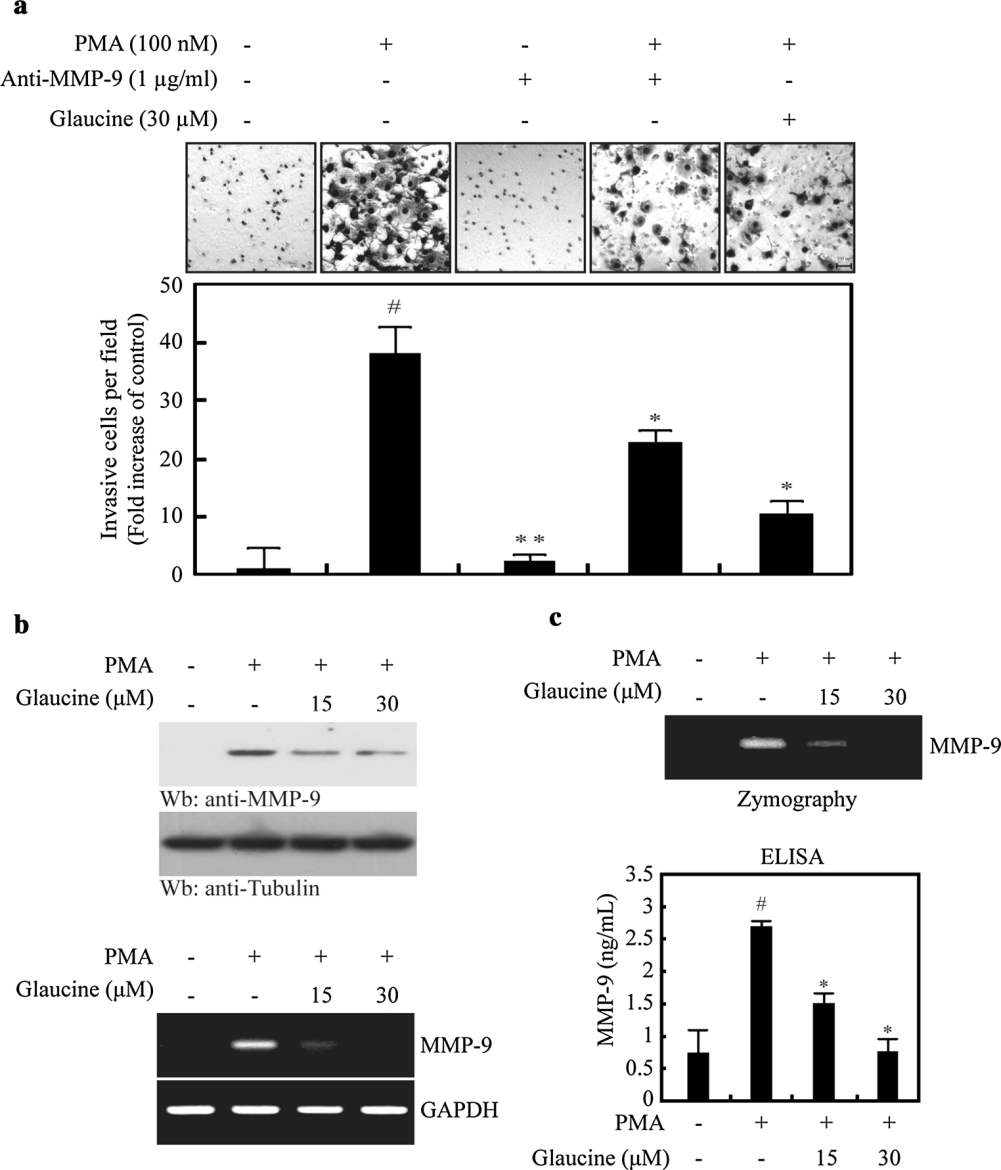
Glaucine reduces PMA-induced expression and secretion of MMP-9 in MCF-7 cells. **a** MCF-7 cells were incubated in Matrigel-coated transwell with or without PMA and anti-MMP-9 antibody or 30 μM glaucine for 36 h. The cell invasion ability was quantified; ^#^ *P* < 0.001 versus vehicle alone-treated cells, ^*^ *P* < 0.01, ^**^ *P* < 0.001 versus PMA alone-treated cells. Data represent the mean ± SE of three independent experiments. **b** MCF-7 cells were incubated with the indicated concentration of glaucine for 30 min followed by 100-nM PMA stimulation for 24 h. After 24 h, the protein level and the mRNA level of endogenous MMP-9 were measured by western blotting (*Top* and 2nd *panels*) and RT-PCR (3rd and *bottom panel*). Tubulin and GAPDH was used as an internal control. **c** MCF-7 cells were pretreated with glaucine for 30 min and stimulated with 100-nM PMA for 24 h. After 24 h, the conditioned medium was collected and assayed for the secreted MMP-9 using gelatin zymography and ELISA; ^#^ *P* < 0.001 versus vehicle alone-treated cells, ^*^ *P* < 0.01 versus PMA alone-treated cells. Data represent the mean ± SE of three independent experiments

MMP-9 is an important ECM-degrading enzyme that has been reported to be involved in cancer cell invasion and migration [5, 6]. To determine whether MMP-9 activity is involved in the PMA-induced invasion of MCF-7 cells, we treated cells with PMA, a primary antibody against MMP-9, or 30 μM glaucine, alone or in combination. Treatment with MMP-9 primary antibody and 30 μM glaucine significantly inhibited PMA-induced cell invasion (Fig. 3a). We also investigated the effects of glaucine on MMP-9 gene expression. As shown in Fig. 3b, PMA induced MMP-9 mRNA and protein expression in MCF-7 cells, whereas treatment with glaucine dose-dependently suppressed PMA-induced MMP-9 gene expression. We next examined the effects of glaucine on the secretion and proteolytic activity of MMP-9 in conditioned media. ELISA and gelatin zymography results revealed that glaucine inhibited PMA-induced MMP-9 secretion and proteolytic activity (Fig. 3c). Collectively, these finding indicate that glaucine suppressed PMA-induced MMP-9 expression at both the protein and mRNA levels and subsequently inhibited MMP-9 enzymatic activity.

### Glaucine inhibits MMP-9 transcriptional activity by suppressing PMA-stimulated NF-κB activity

**Fig. 4:**
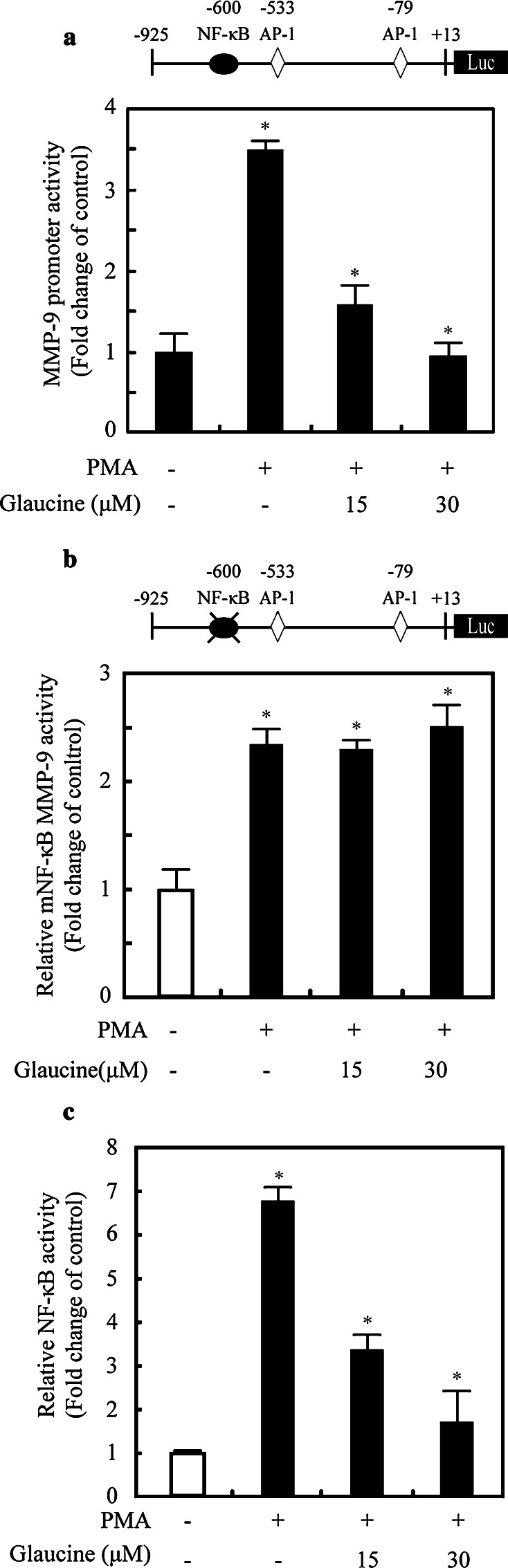
Glaucine inhibits the transcriptional activity of MMP-9 promoter via suppression of PMA-stimulated NF-κB activity. **a** MCF-7 cells were transfected with the pMMP-9-luciferase and the pSV40-β-galactosidase vectors. The transfected cells were treated with the indicated concentrations of glaucine for 30 min and stimulated with 100 nM PMA for 9 h. The luciferase activity was normalized by β-galactosidase activity. Each value represents the mean ± SD of three independent experiments and is expressed relative to a control. **b** MCF-7 cells were transfected with NF-κB binding site mutant (mNF-κB) MMP-9-Luc. The transfected cells were treated with glaucine for 30 min and stimulated with 100 nM PMA for 9 h. The luciferase activity was normalized by β-galactosidase activity. Each value represents the mean ± SD of three independent experiments and is expressed relative to a control. One-way ANOVA was performed to determine statistical significance (^*^ *P* < 0.05). **c** MCF-7 cells were transfected with the reporter plasmids containing tandem NF-κB binding sites. After 24 h, cells were treated with or without 100 nM PMA for 9 h and the luciferase activities were determined. Each value represents the mean ± SD of three independent experiments and is expressed relative to a control. One-way ANOVA was performed to determine statistical significance (^*^ *P* < 0.05)

NF-κB plays an important role in regulating MMP-9 expression in various cancer cell lines [19]. We examined the effects of glaucine on the binding of this transcription factor to the MMP-9 promoter. A luciferase reporter gene containing the MMP-9 promoter region (−925/+13) was transiently transfected into MCF-7 cells, and luciferase activity was determined. MMP-9 promoter activity in cells treated with PMA was upregulated by 3.3-fold compared with that in untreated cells. Glaucine inhibited PMA-induced MMP-9 promoter activity in a dose-dependent manner (Fig. 4a). The transcription factor binding sites in the MMP-9 promoter include sites for NF-κB (−600 bp) [20]. To determine whether NF-κB is involved in MMP-9 transcription regulation, we generated a MMP-9 promoter with a mutation in the NF-κB (−600 bp) binding site. The results of the reporter gene assay showed that PMA-induced promoter activity of the mutated promoter in the NF-κB biding site (-925/+ 13) was not affected by glaucine (Fig. 4b). We also performed experiments using luciferase reporter vectors containing tandem repeats of the NF-κB binding sites and observed dose-dependent reductions in luciferase activity in cells transfected with the NF-κB reporter, following glaucine treatment (Fig. 4c). These results indicated that NF-κB binding to the MMP-9 promoter region contributed to the inhibitory effect of glaucine on PMA-induced MMP-9 transcription.

### Effects of glaucine on NF-κB binding in the MMP-9 promoter

**Fig. 5:**
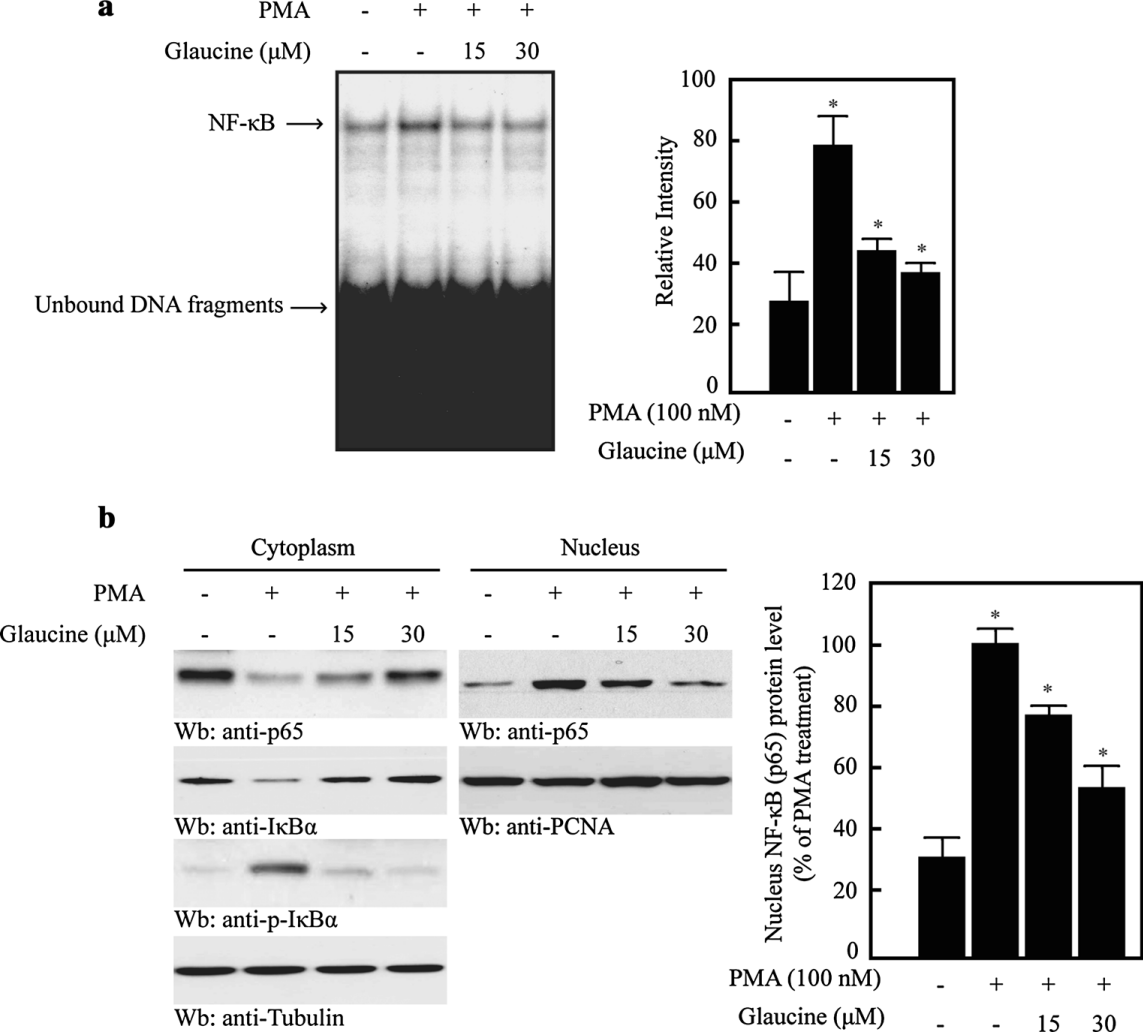
Glaucine inhibits the NF-κB activity in PMA-stimulated MCF-7 cells. **a** MCF-7 cells were pretreated with the indicated concentration of glaucine for 30 min and incubated with 100 nM PMA for 30 min. Nuclear extracts were prepared and incubated with radiolabeled oligonucleotides containing the NF-κB motif in the MMP-9 promoter. **b** MCF-7 cells were pretreated with the indicated concentration of glaucine for 30 min and incubated with 100-nM PMA for 30 min. Cells were harvested and fractionated into the cytoplasm and the nucleus. Lysates were then separated on a 10 % SDS–polyacrylamide gel and subjected to western blotting with anti-p65, anti-p-IκBα, and anti-IκBα antibodies. The analysis was repeated in three times, and α-tubulin and PCNA were used as markers for the cytoplasmic and nuclear fractions. The NF-κB protein level that was translocated to the nucleus was quantified by densitometric analyses. The bars represent the mean ± SD. One-way ANOVA was performed to determine statistical significance (^*^ *P* < 0.05)

We next performed EMSAs to determine the effect of glaucine on the DNA-binding activity of NF-κB. As shown in Fig. 5a, PMA induced NF-κB DNA-binding activity, which was dose-dependently reduced by glaucine. To confirm this observation, we analyzed p65 levels in the cytosolic and nuclear fractions. The results showed that PMA increased IκBα phosphorylation in the cytoplasm, leading to increased nuclear translocation of the NF-κB p65 subunit (Fig. 5b). However, glaucine inhibited PMA-induced phosphorylation of IκBα in a dose-dependent manner (Fig. 5b) and decreased nuclear NF-κB p65 subunit levels (Fig. 5b, right panel). These results indicate that glaucine inhibits NF-κB activation by suppressing IκBα phosphorylation and the subsequent nuclear translocation of NF-κB in PMA-treated MCF-7 cells.

### Glaucine suppresses MDA-MB-231 cells invasion by inhibiting MMP-9 through the NF-κB pathway

**Fig. 6:**
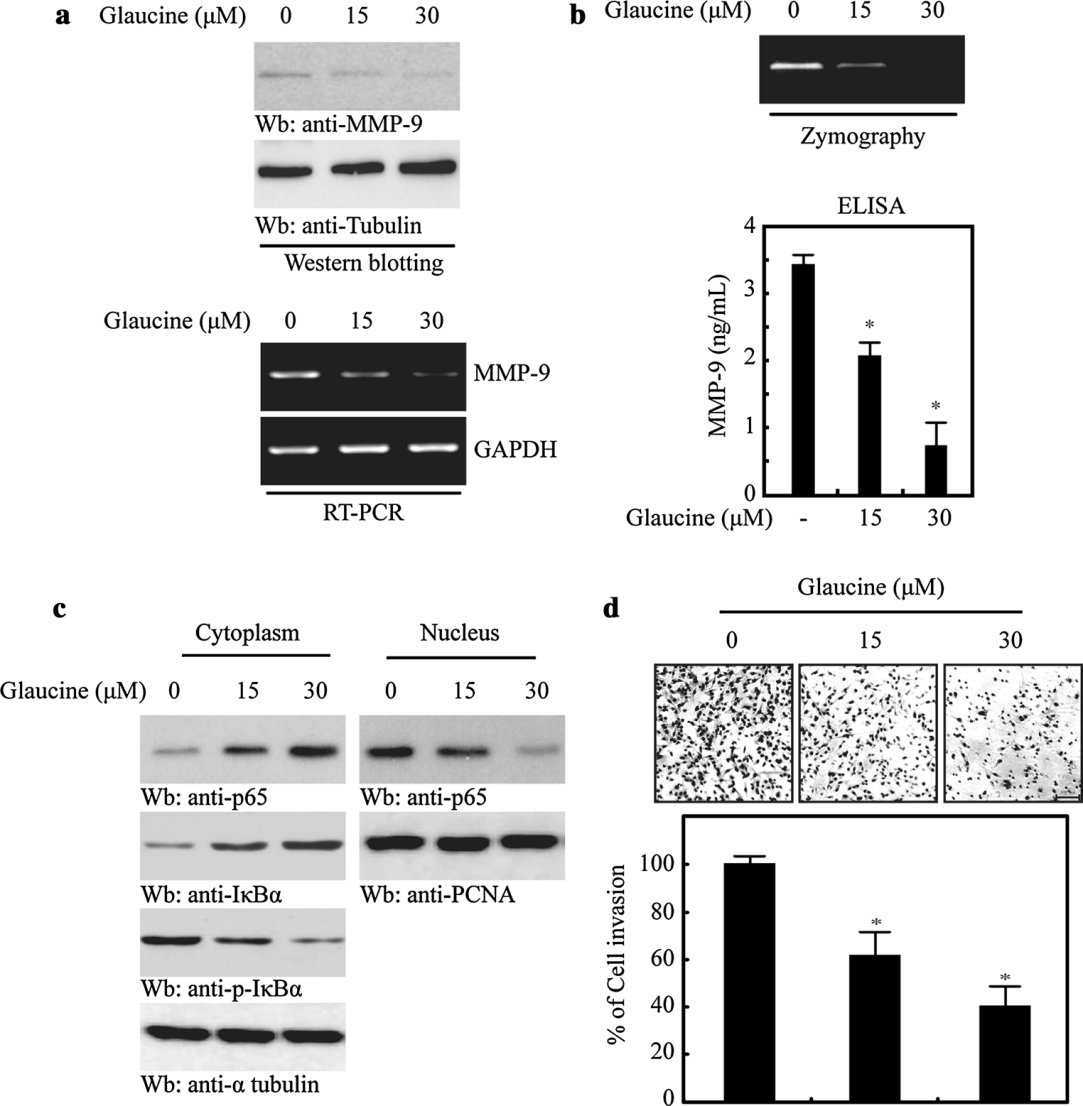
Glaucine inhibits the invasion of MDA-MB-231 cells by inhibiting MMP-9 expression and secretion through the NF-κB pathway. **a** MDA-MB-231 cells were treated with the indicated concentration of glaucine for 24 h. After 24 h, the protein level and the mRNA level of endogenous MMP-9 were determined by western blotting (*Top* and 2nd *panels*) and RT-PCR (*third* and *bottom panel*). Tubulin and GAPDH was used as an internal control. **b** MDA-MB-231 cells were treated with the indicated concentration of glaucine for 24. After 24 h, the conditioned medium was collected and assayed for the secreted MMP-9 using gelatin zymography and ELISA; ^*^ *P* < 0.01 versus vehicle control cells. Data represent the mean ± SE of three independent experiments. **c** MDA-MB-231 cells were treated with the indicated concentration of glaucine for 4 h. The cells were harvested and fractionated into the cytoplasm and the nucleus. Lysates were then separated on a 10 % SDS–polyacrylamide gel and subjected to western blotting with anti-p65, anti-p-IκBα, and anti-IκBα antibodies. The analysis was repeated in three times, and α-tubulin and PCNA were used as markers for the cytoplasmic and nuclear fractions. **d** MDA-MB-231 cells were treated with the indicated concentration of glaucine for 36 h. The invasive ability of cells determined using Matrigel-coated transwell. The cell invasion ability was quantified; ^*^ *P* < 0.001 versus vehicle alone-treated cells. Data represent the mean ± SE of three independent experiments

The results obtained in MCF-7 cells suggested that glaucine inhibits PMA-induced MMP-9 expression by suppressing the nuclear translocation of NF-κB. To demonstrate the functional consequence of glaucine in metastatic breast cancer, we used it to treat MDA-MB-231 cells, a highly metastatic breast cancer cell line that highly expresses MMP-9 and constitutively activated NF-κB [21, 22]. MDA-MB-231 cells were incubated with glaucine (0–30 μM), and we monitored changes in MMP-9 expression. As shown in Fig. 6a, treatment with glaucine for 24 h decreased MMP-9 expression in a dose-dependent manner, as measured by western blotting and RT-PCR. We further assessed the MMP-9 secretion in glaucine-treated MDA-MB-231 cells by zymography and ELISA. The results revealed that glaucine inhibited MMP-9 secretion and proteolytic activity (Fig. 6b). These findings support the results we obtained in PMA-induced MCF7 cells (Fig. 3). To assess the effects of glaucine on NF-κB translocation further, we performed western blots of different subcellular fractions to show that p65 nuclear translocation was substantially augmented after glaucine treatment (Fig. 6c top panel). Finally, we performed invasion assays and found that glaucine significantly inhibited MDA-MB-231 cell invasion in a dose-dependent manner (Fig. 6d). Taken together, our findings demonstrate that glaucine inhibits breast cancer cell migration and invasion by inhibiting NF-κB-mediated MMP-9 transcription.

## Discussion

For centuries, people have been harnessing the power of nature to provide medicinal solutions to various diseases. Indeed, in the context of cancer, plants in particular are the rich sources of natural compounds that possess anticancer activity and are used as novel lead compounds and chemical entities to derive superior new compounds. In this study, we investigated whether glaucine could inhibit human breast cancer cell migration and invasion. We demonstrated that: (a) glaucine inhibits migration and invasion, (b) regulates MMP-9 expression and activity, and (c) specifically abrogates NF-κB activation in both PMA-treated MCF-7 cells and MDA-MB-231 cells.

Cancer invasion and metastasis are major properties of various malignant tumors and the main causes of treatment failure. A vital step in the invasive processes is the proteolytic degradation of the ECM by proteolytic enzymes such as MMPs [5, 6]. In particular, MMP-9 overexpression has been associated with the progression and invasion of different types of tumors, including mammary tumors [23]. Thus, agents and/or natural plant products that inhibit both MMP-9 expression and activity have received considerable attention for their potential use in the treatment of malignant and invasive cancers [8].

Our results indicate that glaucine is a potent inhibitor of PMA-induced MMP-9 expression in two breast cancer cell lines (MCF-7 and MDA-MB-231), suggesting that the ability of glaucine to inhibit MMP-9 expression may be a general phenomenon. western blotting, RT-PCR, and luciferase reporter gene assays revealed that the target of the inhibitory effect of glaucine was MMP-9 gene transcription.

As described in a previous study, we found that PMA stimulated MMP-9 expression and secretion [24, 25]. This is in accordance with our observation that glaucine inhibited the PMA-induced secretion of MMP-9 in a dose-dependent manner by suppressing the transcriptional activity of the MMP-9 gene in MCF-7 cells. Glaucine also dose-dependently inhibited the enzymatic activity of MMP-9 secreted from PMA-induced MCF-7 cells, suggesting that it is a strong candidate for decreasing tumor metastasis and invasion via dual inhibition of MMP-9 gene transcription and enzyme activity.

The MMP-9 promoter region contains the NF-κB binding site [25], and we showed that this site was necessary for glaucine’s ability to inhibit MMP-9 expression in both PMA-treated MCF-7 cells and MDA-MB-231 cells which have constitutively activated NF-κB. This protein complex is closely linked to inflammation, tumor cell proliferation, survival, invasion, and metastasis [26, 27]. Notably, NF-κB is constitutively activated in many cancers and contributes to resistance to chemo- and radiotherapies by promoting the expression of anti-apoptotic proteins [28]. Here, we demonstrated that glaucine suppressed MMP-9 expression by inhibiting NF-κB activity in PMA-treated MCF-7 and MDA-MB-231 cells.

In conclusion, we provide novel evidence that glaucine suppresses breast cancer cell invasion and migration by inhibiting MMP-9 activity and expression, which was due to the blocking of NF-κB activation. An increasing number of natural products possessing anticancer properties have been unveiled in recent years. Once their antitumor mechanisms are elucidated, these compounds could be used in clinical chemotherapeutic strategies. In view of the impressive in vitro potency of glaucine in reversing PMA-induced cancer cell invasion at non-cytotoxic concentrations (data not shown) and the fact that specific anti-metastatic agents with minimal or no complications due to cytotoxicity are preferred, naturally occurring phytochemicals are indeed worthy of further development as anti-metastatic agents for clinical use.
